# Dr. Edward B. Belknap

**Published:** 1913-07

**Authors:** 


Dr. Edward B. Belknap, Buffalo 1892, of Wyoming, died at the Rochester General Hospital, May 12, of nephritis, aged 46.

